# Influence of pH and protein concentration on the development of myofibrillar protein-based biodegradable film from Basa (*Pangasius bocourti*) fish waste

**DOI:** 10.3389/fnut.2026.1768718

**Published:** 2026-08-14

**Authors:** Prashant K. Mishra, Mohd Danish, Vivekanand Kumar, Aarti S. Kakatkar, Raj Kamal Gautam, Suchandra Chatterjee

**Affiliations:** 1 Food Technology Division, FIPLY, Bhabha Atomic Research Centre, Mumbai, Maharashtra, India; 2 Centre of Food Technology, University of Allahabad, Prayagraj, Uttar Pradesh, India; 3 Homi Bhabha National Institute, Mumbai, Maharashtra, India

**Keywords:** biodegradable film, fish myofibrillar protein, fish waste, *Pangasius bocourti*, pH

## Abstract

Fish waste generated by fish-processing industries contributes significantly to environmental pollution, highlighting the need for its effective utilization. Similarly, plastic waste from packaging industries poses serious environmental and health concerns. In this study, a biodegradable film was developed using myofibrillar protein obtained from Basa (*Pangasius bocourti*) fish waste. Protein concentrates at different concentrations (0.5, 1.0, and 1.5%) were used to prepare films under two pH conditions (2.8 and 11.0). The films were evaluated for their tensile strength, elongation at break, water vapor permeability, water solubility, and color characteristics. Among the formulations tested, the film prepared with 1% protein concentrate at pH 2.8 exhibited the highest tensile strength (6.86 MPa), the lowest water solubility (28.1%), and a lower increase in yellowness during storage compared to the other films. Films developed under acidic conditions showed greater disulphide interactions and a smoother surface morphology than those prepared under alkaline conditions. Thus, a myofibrillar protein-based biodegradable film was developed and it was systematically demonstrated how pH and protein concentration affect their mechanical, barrier, and physicochemical properties. The innovation of this work lies in integrating Basa fish waste valorization with pH-mediated protein engineering to develop biodegradable films.

## 1 Introduction

Fish is highly nutritious and rich in essential micronutrients and macronutrients. There is a demand for fish worldwide. Hence, there is a requirement for higher fish production and processing, which would lead to a higher generation of fishery waste. Therefore, it is important to reduce this waste (1). Waste generated from fish processing accounts for 60–70% of fresh fish weight (2). This waste is generally discarded in an open environment, which is a serious environmental concern. This waste is also a rich source of many important compounds. Hence, the effective utilization of fishery waste could produce value-added products and control pollution. There are studies on the utilization of fish waste to develop useful products (2).

There is a high demand for plastics due to their superior properties, and over half of produced plastics is used in packaging, particularly in the food industry, because packaging preserves the food quality, which leads to lower food waste (3). Global plastic production was 450 million metric tons in 2025 (4), while plastic waste generation was 225 million metric tons (28 kg/capita/year) (5). However, plastic waste causes pollution due to its non-biodegradability by accumulating in ecosystems. Hence, there is a requirement for new food packaging materials. To solve the issue, biodegradable packaging is one of the alternatives that may be useful in reducing pollution. Biodegradable packages/films are prepared from biodegradable and non-toxic biomass (6, 7).

India ranks second in global fishery production and actively exports 11% of its catch. This includes shrimp, dried fish, frozen fish, chilled fish, as well as other processed fish products. Fish processing includes beheading, degutting, deskinning, and deshelling, which generates huge waste by the seafood industry. Shellfish waste accounts for 40–50% of total fish waste, amounting to approximately 80,000 metric tons in India. This waste includes fins, bones, skin, mince, and viscera, which collectively contain high amounts of nutrients, with 60% protein, 19% fat, and 22% ash content, and leads to huge economic and environmental losses. Minimization of this loss is one of India’s goals for sustainable development, and India has adopted a “more for less” policy, which emphasizes recycling and the reuse of available sources for waste minimization (8).

Basa fish is high in demand due to its easily digestible proteins, good taste, and white flesh and is mainly processed as boneless fillets (8). This leads to substantial waste generation, and this waste also contains fish mince rich in myofibrillar proteins. Our lab previously reported on the utilization of this fish waste to prepare biodegradable films, fish oil, and pet food (8–10).

Myofibrillar proteins have excellent film-forming properties with lower water solubility which is good for biodegradable package formation. These proteins, though insoluble in water, can be solubilized by regulating the pH of the solution. This fully stretches the proteins and links them in parallel structures. This results in the formation of a continuous matrix of proteins during the drying of the solution (9).

There are previous reports on the development of films using fish myofibrillar proteins. For instance, the effect of pH modifications was studied on white croaker fish myofibrillar proteins (11), active film was prepared using *Scomberomorus brasiliensis* fish myofibrillar protein waste with passion fruit essential oil (12), water resistant films were prepared from silver carp fish myofibrillar proteins by integrating polyphenol with cellulose nanocrystals (13), and effect of protein concentrations were studied on the development of tilapia fish myofibrillar protein films (14).

Thus, this study aims to identify the effect of pH and protein concentrations on the development of biodegradable film using myofibrillar protein obtained from basa (*Pangasius bocourti*) fish waste.

## 2 Materials and methods

### 2.1 Chemicals

Glacial acetic acid, sodium potassium tartrate sodium carbonate, and Folin–Ciocalteu reagent were purchased from Loba Chemie, India. Sodium chloride (NaCl) and sodium hydroxide (NaOH) were purchased from Polypharm Private Limited, India. Tris(hydroxymethyl)aminomethane (Tris), Disodium ethylenediaminetetraacetic acid (Na_2_EDTA), glycine, urea, and sodium dodecyl sulfate (SDS) were purchased from Himedia, India. 2-mercaptoethanol (2-ME) and bovine serum albumin (BSA) were purchased from Sigma Aldrich, USA.

### 2.2 Preparation of protein dry concentrate (PDC) from fish waste

Basa fish waste was procured from a fish-processing industry located in Mumbai and transported on ice to the laboratory, followed by mince separation from the waste using a fish deboning machine (stainless steel, belt-and-drum-type, perforated drum with pore size of 4 mm diameter, Rajma Engineering Company, India). The mince was washed as described in a previous study with minor modifications (14). Initially, the mince was washed using chilled distilled water, followed by centrifugation (Thermo Scientific, Heraeus multifuge X3R, USA). The pellet obtained was then washed with 50 mM NaCl solution (1,3 w/v) followed by centrifugation. This pellet was freeze dried (Christ, Beta 1–8 LO plus, Germany) and crushed using a grinder (Sumeet, CAT:950, India) to obtain dry concentrate (PDC).

### 2.3 Proximate composition of PDC

The protein content of PDC was determined using Kjeldahl (VELP Scientifica Srl, UDK 159, Italy). Moisture content was determined using a moisture analyzer (OHAUS, MB45, USA) at 110 °C. Fat content was determined using the Soxhlet apparatus (Borosil Scientific, SOX065, India) and ash content was determined using a muffle furnace (Meta Lab Scientific industries, MSI-50A, India) as described by the AOAC method (15). Carbohydrate content was calculated using the difference method.

### 2.4 Preparation of film-forming dispersion

Initially, PDC was dispersed at different pH solutions (2 – 12), in which the highest protein solubility was found at pH 2.8 and 11. Hence, further experiments were done at these pH conditions. The film-forming dispersion was prepared as per a previous report (16). Dispersion was prepared using lyophilized PDC at 0.5, 1, and 1.5% by dry weight in distilled water (w/v). Glycerol (50% by weight of PDC, w/w) was added as a plasticizer followed by pH adjustment (Thermo Scientific, Eutech expert pH, USA) of dispersions to 2.8 using glacial acetic acid and 11 using NaOH. The dispersions were kept in a shaking incubator (Remi, Cis-24 plus, India) at 25 °C for 16 h followed by homogenization (Kinemetica, polytron PT3100, Switzerland).

### 2.5 Film preparation

Different films were prepared by pouring film-forming dispersion (50 mL) on a polystyrene Petri dish (85 mm dia, Laxbro, India) followed by drying at 50 °C for 24 h at 50% relative humidity (RH) in a stability chamber (Patel Scientific Instruments, PSI 3000, India). The films were conditioned for 24 h at 25 °C at 50% RH in a stability chamber.

### 2.6 Measurement of mechanical property

The mechanical properties of films such as tensile strength (TS) and elongation at break (EB) were calculated as per previous report (9). Briefly, films were cut to dimensions of 70 mm length × 20 mm width. The thickness of films were measured using a digital micro-meter (Mitutoyo, MDC-25SB, Japan). The cut films were used to measure TS and EB using a TA texture analyser (TA. HD. Plus, Stable Microsystems, UK). The initial grip separation used was 50 mm and the rate of grip separation used was 500 mm/min. TS and EB were calculated as per the following formulae:

$$\mathrm{TS}\;(\mathrm{MPa})=\frac{\text{Maximum force required to break film}\;(N)}{\text{width of film}\;(\mathrm{mm})\times \text{thickness of film}\;(\mathrm{mm})}$$

$$\mathrm{EB}\;(\%)=\frac{\text{Length of film}\;\mathrm{at}\;\text{break}\;(\mathrm{mm})-\text{Initial length of film}(\mathrm{mm})}{\text{Initial length of film}\;(\mathrm{mm})}\times 100$$

### 2.7 Measurement of water vapor permeability and water solubility

The water solubility (WS) and water vapor permeability (WVP) of films at different temperatures (25–65 °C) were evaluated as reported previously (9). In short, to measure WS, film discs (2 cm diameter) were immersed in distilled water (50 mL) for 24 h at different temperatures. The film discs were dried at 105 °C and weighed before and after immersion in distilled water till constant weight using weighing balance (Shimadzu Corporation, ATX224, Japan). WS of films were determined using the following equation:

$$\begin{array}{l} \mathrm{WS}\;(\%)\\ =\frac{\text{Initial weight of dried film}\;(\mathrm{mg})-\text{Final weight of dried film}\;(\mathrm{mg})}{\text{Initial weight of dried film}\;(\mathrm{mg})}\times 100 \end{array}$$

To measure WVP, films were fixed firmly on the opening of cells containing dried silica and kept inside a desiccator equilibrated with distilled water at different temperatures and periodic change in weight of cells was measured. WVP was calculated as per the following equation:

$$\mathrm{WVP}\;(10^{-8}g\;\mathrm{mm}\;h^{-1}\;{\mathrm{cm}}^{-2}\;{\mathrm{Pa}}^{-1})=(\frac{w}{t})\times (\frac{x}{A\Delta P})$$

where w/t is the slope obtained from the weight gain of the cell versus time plot (g·h^−1^), x is the thickness of the film (mm), A is the permeation area (cm^2^), and ∆P is the difference in partial pressure between the silica gel-containing atmosphere and pure water (Pa).

### 2.8 Determinations of protein solubility in different solvent

To determine the interactions involved during film preparation, films and PDC were incubated with different solutions as per previous report (17). A sample (5 mg) was dispersed in 1 mL of different solvents: deionized water; Tris-Glycine buffer (0.086 M Tris, 0.09 M glycine, and 4 mM Na_2_EDTA, pH 8.0); and Tris-Glycine buffer containing 0.5% sodium dodecyl sulfate (SDS) and 8 M urea (d) Tris-Glycine- urea (8 M)-SDS (0.5%) buffer containing 1% (v/v) 2-mercaptoethanol (2-ME). The samples were kept in a shaker (Remi, Cis-24 plus, Mumbai, India) at 25 °C for 16 h followed by centrifugation (Thermo Scientific, Heraeus multifuge X3R, USA) at 12000 rpm for 10 min at 25 °C. Protein concentration of the supernatant was determined by the Lowry method using BSA as a standard by taking absorbance (Shimadzu corporation, UV1900, Japan) at 750 nm (18).

### 2.9 Color analyses of films with storage

CIELAB parameters, namely L* (lightness), a* (+red to –green component), and b* (+yellow to –blue component) of films, were determined using a colorimeter (Konica Minolta Sensing Inc., CM-3600d, Japan). The films were stored at room temperature (25 °C), and the change in color parameters was measured for 15 days.

### 2.10 Surface microstructure of film

The surface morphologies of the films were studied using scanning electron microscope (SEM) (Seron Technologies Inc., ATS 2100, Republic of Korea). Film pieces were coated with a thin layer of gold (<20 nm) to prevent charging during electron beam exposure. Images were taken in 20 keV.

### 2.11 Statistical analysis

DSAASTAT ver. 1.101 by Andrea Onofri was used for the statistical analysis of data (19). The obtained values (*n* = 9) were expressed as average ± standard deviation. Data were analyzed by two-way analysis of variance (ANOVA), and multiple comparisons of means were carried out using Duncan’s multiple range tests at *p* < 0.05.

## 3 Results

### 3.1 Proximate composition of PDC

The protein, fat, carbohydrate, moisture, and ash content of PDC was 87.6 ± 0.3%, 6.0 ± 0.1, 0.4 ± 0.03, 3.8 ± 0.2, and 2.2 ± 0.1%, respectively, in the present study.

### 3.2 Mechanical properties, WS, and WVP of films

Mechanical properties (thickness, TS, and EB) are given in Table 1a. WS and WVP of the films prepared using different PDCs at pH 2.8 and 11 are given in Table 1b.

**Table 1:** Mechanical properties [thickness, TS, and EB (a)] and [WS, and WVP (b)] of films developed at pH 2.8 and 11.

(a)	Thickness (mm)	TS (MPa)	EB (%)
Films at pH 2.8			
0.5% PDC	0.049 ± 0.004^b^	3.86 ± 0.07^d^	58.56 ± 2.39^b^
1% PDC	0.112 ± 0.008^d^	6.86 ± 0.08^f^	146.76 ± 5.71^d^
1.5% PDC	0.165 ± 0.008^f^	4.86 ± 0.08^e^	87.18 ± 4.09^c^
Films at pH 11			
0.5% PDC	0.039 ± 0.002^a^	2.43 ± 0.09^a^	42.78 ± 2.36^a^
1% PDC	0.082 ± 0.003^c^	2.78 ± 0.08^b^	228.46 ± 12.33^e^
1.5% PDC	0.142 ± 0.004^e^	3.25 ± 0.07^c^	147.84 ± 6.33^d^

(b)	WS (%)	WVP (10^−8^ g mm h^−1^ cm^−2^ Pa^−1^)
Films at pH 2.8		
0.5% PDC	27.6 ± 0.7^a^	2.93 ± 0.10^a^
1% PDC	28.1 ± 0.4^a^	3.29 ± 0.05^b^
1.5% PDC	31.2 ± 0.8^b^	5.97 ± 0.08^d^
Films at pH 11		
0.5% PDC	35.0 ± 0.8^c^	3.25 ± 0.05^b^
1% PDC	37.6 ± 0.7^d^	3.86 ± 0.08^c^
1.5% PDC	39.3 ± 0.7^e^	6.55 ± 0.11^e^

The results shown are Mean ± Standard deviation of experiments (*n* = 9). The different letters in columns indicate significant differences at *p* < 0.05.

### 3.3 Protein solubility of different films and PDC in different solvents

**Figure 1:**
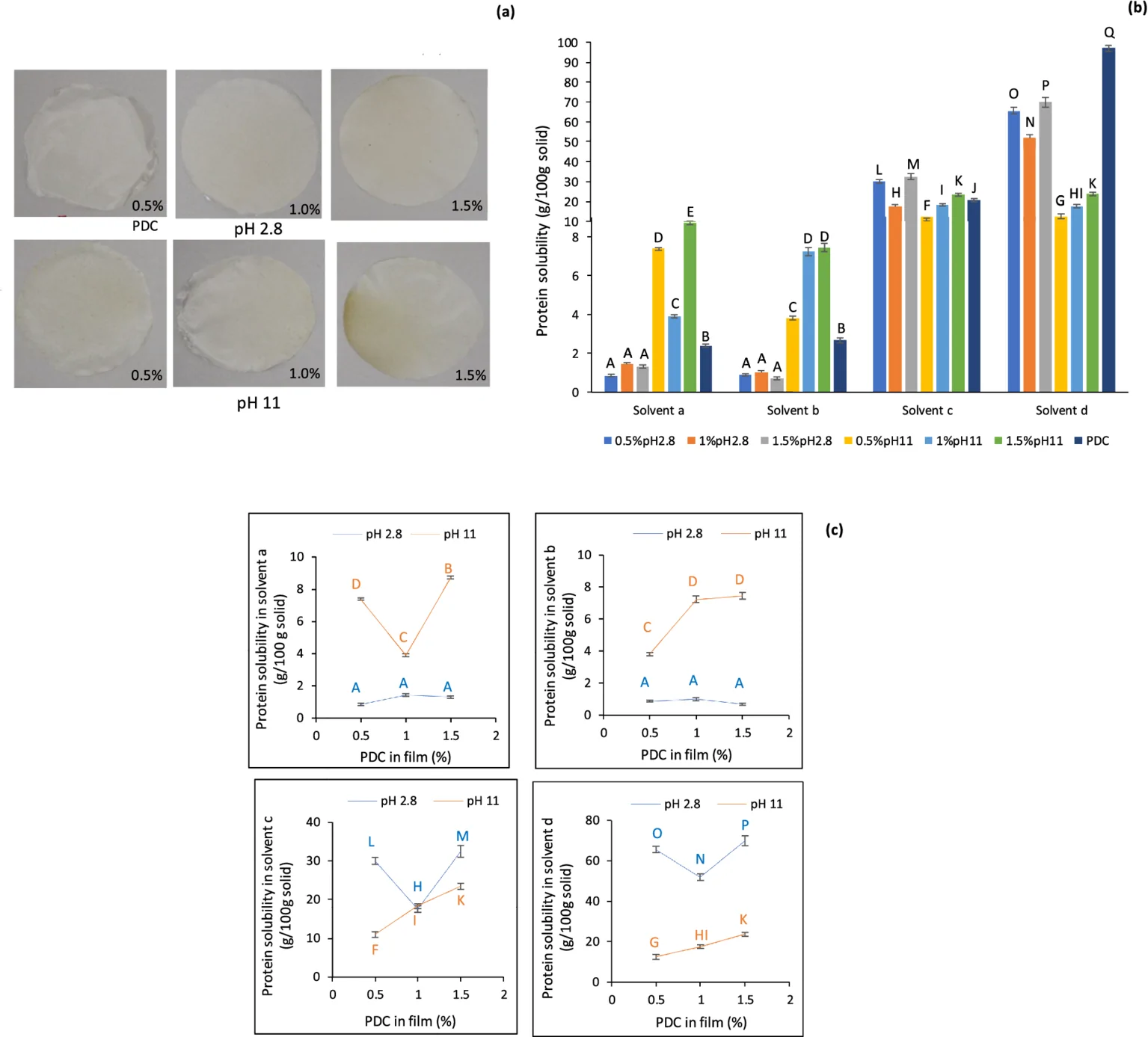
**(a)** Appearance of different films, **(b)** protein solubility of films and protein dry concentrate in different solvents, and **(c)** protein solubility of films in different solvents with PDC concentration. The results of panel **(b,c)** are mean ± standard deviation of experiments (*n* = 9). The different letters on error bars indicate significant differences at *p* < 0.05.

The appearance of different films is shown in Figure 1a. The protein solubility of different films as well as PDC in different solvents is shown in Figure 1b and the protein solubility of films with PDC concentration in different solvents is shown in Figure 1c.

### 3.4 Color of films

The color properties of films with storage are given in Table 2.

**Table 2:** Color properties L* (a), a* (b), and b* (c) of film prepared at pH 2.8 and 11 with storage days.

(a)	L* with storage days			
	Day 0	Day 5	Day 10	Day 15
Films at pH 2.8				
0.5% PDC	41.5 ± 0.2^fghi^	41.8 ± 0.9^ijk^	40.9 ± 0.1^bc^	40.7 ± 0.07^b^
1% PDC	41.3 ± 0.7^defgh^	41.9 ± 0.01^jkl^	42.2 ± 0.1^l^	41.4 ± 0.06^defgh^
1.5% PDC	41.4 ± 1.0^efgh^	42.2 ± 0.05^kl^	41.6 ± 0.02^ghij^	41.2 ± 0.02^cdef^
Films at pH 11				
0.5% PDC	41.2 ± 0.2^cdef^	41.7 ± 0.5^hij^	41.0 ± 0.01^bcde^	40.7 ± 0.08^b^
1% PDC	41.2 ± 0.01^cdefg^	42.1 ± 0.4^kl^	40.9 ± 0.5^bcd^	40.9 ± 0.08^bcd^
1.5% PDC	41.1 ± 0.5^cdef^	41.5 ± 0.3^fghi^	41.0 ± 0.01^bcde^	40.2 ± 0.08^a^

(b)	a* with storage days			
	Day 0	Day 5	Day 10	Day 15
Films at pH 2.8				
0.5% PDC	−6.6 ± 0.2^a^	−4.0 ± 0.3^b^	−1.0 ± 0.008^g^	0.1 ± 0.01^jk^
1% PDC	−2.5 ± 0.1^e^	−0.5 ± 0.04^h^	0.09 ± 0.009^jk^	0.2 ± 0.01^klm^
1.5% PDC	2.9 ± 0.2^r^	3.1 ± 0.04^s^	3.2 ± 0.02^s^	3.7 ± 0.03^t^
Film at pH 11				
0.5% PDC	−2.2 ± 0.1^f^	0.3 ± 0.03^mn^	0.5 ± 0.03^op^	0.6 ± 0.03^q^
1% PDC	−2.7 ± 0.1^d^	0.15 ± 0.01^jkl^	0.4 ± 0.02^no^	0.5 ± 0.02^p^
1.5% PDC	−3.2 ± 0.2^c^	−0.02 ± 0.002^i^	0.04 ± 0.004^ij^	0.2 ± 0.02^lm^

(c)	b* with storage days			
	Day 0	Day 5	Day 10	Day 15
Films at pH 2.8				
0.5% PDC	−1.2 ± 0.09^a^	0.6 ± 0.02^d^	0.9 ± 0.03^f^	1.2 ± 0.03^g^
1% PDC	−0.7 ± 0.05^b^	0.8 ± 0.08^e^	1.4 ± 0.05^h^	1.5 ± 0.01^i^
1.5% PDC	1.7 ± 0.1^j^	2.0 ± 0.08^k^	2.8 ± 0.05^n^	3.1 ± 0.06^o^
Film at pH 11				
0.5% PDC	−1.3 ± 0.08^a^	0.5 ± 0.04^c^	0.8 ± 0.02^e^	2.1 ± 0.04^l^
1% PDC	0.4 ± 0.02^c^	1.2 ± 0.05^g^	3.6 ± 0.1^p^	4.2 ± 0.05^q^
1.5% PDC	2.5 ± 0.1^m^	4.8 ± 0.09^r^	6.3 ± 0.05^s^	7.0 ± 0.07^t^

The results shown are Mean ± Standard deviation of experiments (*n* = 9). The different letters in columns indicate significant differences at *p* < 0.05.

### 3.5 Surface micrograph, WS, and WVP of films with 1% PDC

**Figure 2:**
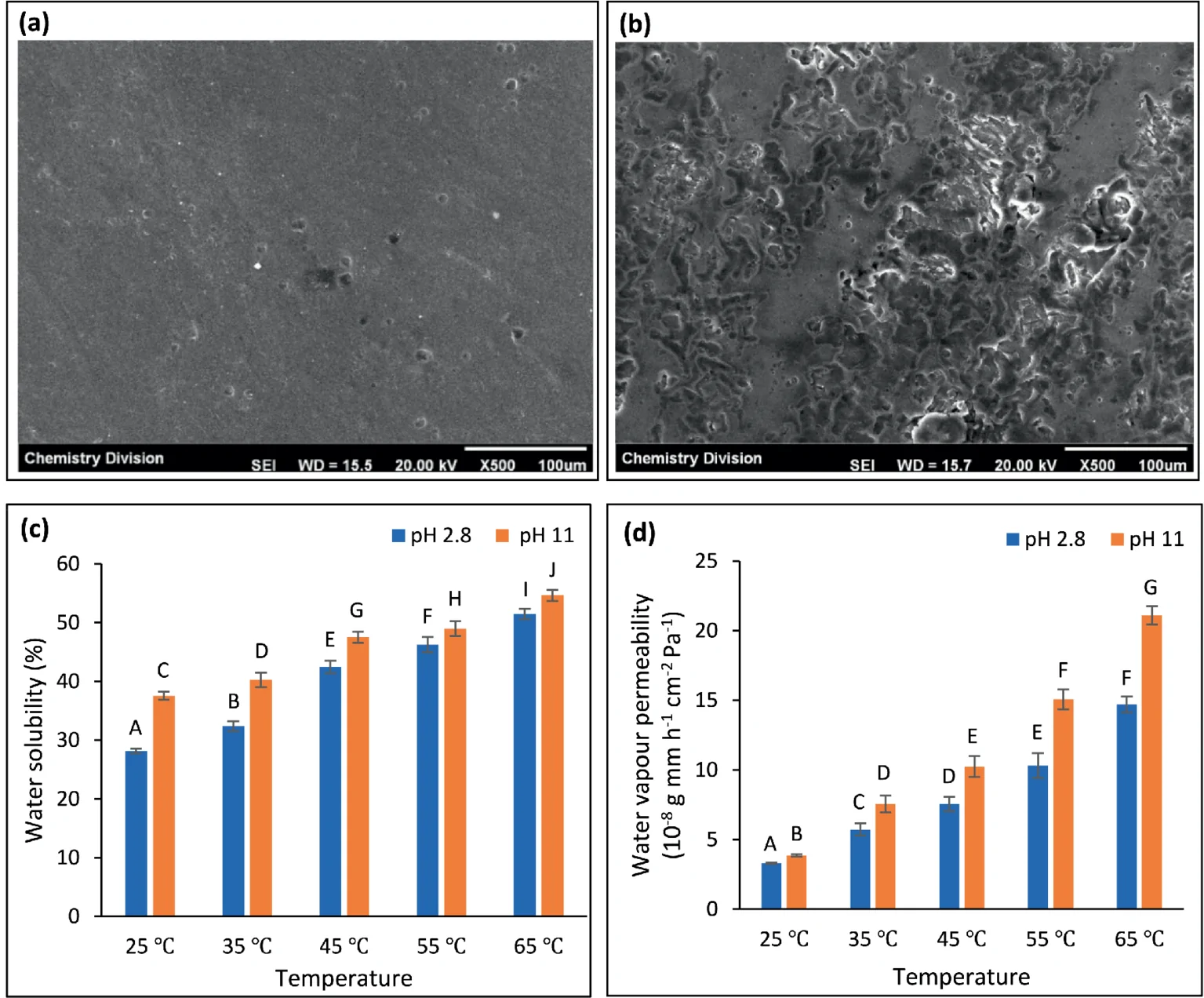
SEM micrograph of 1% protein concentrate film developed at pH 2.8 **(a)**, and 11 **(b)**, water solubility at different temperature **(c)**, and water vapor permeability at different temperature **(d)** of 1% protein concentrate film developed at pH 2.8 and 11. The results of panel (c,d) shown are mean ± standard deviation of experiments (*n* = 9). The different letters on error bars indicate significant differences at *p* < 0.05.

Surface micrographs of films developed using 1% PDC at pH 2.8 and 11 are shown in Figures 2a,b respectively. WS and WVP of films at different temperature developed using 1% PDC are shown in Figures 2c,d respectively.

## 4 Discussion

### 4.1 Proximate composition of PDC

The protein and ash content of PDC were similar to those reported in a previous study (20). The moisture content found in Burrito (*Brachydeuterus auritus*) fish powder and Tuna trimming powder was similar to our results (21). The fat content of powder obtained from Herring was similar to the results in the present study (22). The carbohydrate content of powder obtained from Burrito fish was 1% (21). The proximate composition of PDC obtained from fish depends on the type of fish, developmental stage, gender, and method of extraction.

### 4.2 Mechanical properties, WS, and WVP of films

The thickness of films increased (p < 0.05) with increasing concentration of PDC at both pH conditions (Table 1). A previous report also showed an increase in the thickness of films as the concentration of protein increased at both acidic and alkaline pH conditions (23). Higher thickness of films at higher PDC is due to an increase in the solid content of films after drying (24).

Generally, TS increases with the increasing concentration of protein, as the number of protein chains increases per surface unit, leading to higher intermolecular interactions (14). However, in the present study, TS first increased (*p* < 0.05) and then decreased (*p* < 0.05) with increasing PDC concentrations at pH 2.8. However, at pH 11, there was no effect (*p* > 0.05) on TS, as PDC increased from 0.5 to 1%, followed by an increase (*p* < 0.05) in TS as concentration increased (Table 1). A previous report also showed an increase in TS as chitosan concentration increased in corn starch-chitosan film followed by a decrease in TS similar to the present study for the films which were prepared under acidic conditions. The reason for the decrease in TS at higher protein concentrations may be formation of non- homogenous film forming solution (25). However, films developed at an alkaline pH showed an increase in TS with increasing concentration of PDC similar to previous report (26). Films developed under acidic condition had better tensile strength than films developed under alkaline condition. Previous studies have also reported higher TS of films developed using tilapia myofibrillar protein under acidic conditions than those prepared under alkaline conditions. The authors reported that acidic pH might favor the solubilization and subsequent alignment of protein molecules, in such a way through which strong bond interjunctions were formed (27). Under acidic conditions, protonation of charged amino acids leads to higher changes in secondary structure of myofibrillar proteins as compared to alkaline condition (28). Hence, there is more unfolding of proteins leading to the development of more new interactions during drying of film-forming solutions causing higher TS of films in present case.

As per a previous report, an increase in the protein concentration of films leads to higher EB due to greater intermolecular aggregation of protein, compared with films with lower protein concentration, resulting in improved flexibility (14). In the present study also, the EB was found to increase (*p* < 0.05) initially followed by decrease (*p* < 0.05) as PDC concentration increased under both pH conditions (Table 1). As the concentration of chitosan was increased in the film developed from chitosan/starch film, EB was found to initially increase and then decrease, similar to the present study. The reason for the decrease in EB at higher concentrations may be formation of non-homogenous film-forming solution (25). A previous report also showed decrease in EB of films with increasing concentration of protein and suggested that EB is inversely related to the thickness of film similar to present study in case of 1.5% PDC (24).

There was no significant change in WS with increasing concentrations of PDC up to 1%, however an increase in WS at 1.5% PDC for films developed under acidic conditions. Whereas an increase in WS as PDC concentration increased in films developed under alkaline conditions was observed (Table 1). A previous report also showed an increase in WS as the concentration of white mouth croaker myofibrillar protein increased, similar to the present study (24). The presence of high concentrations of glycerol with increasing concentrations of PDC (as glycerol content is 50% of the PDC concentration in the present study) is the reason for increased WS in relation to PDC concentration, as glycerol has a hydrophilic nature (29). In the present study, higher WS was found in alkaline conditions as compared to acidic conditions as reported previously for WS of bigeye snapper fish myofibrillar protein films. The formation of higher disulphide interactions during film formation at an acidic pH might be the reason for lower WS as compared to films developed at an alkaline pH (30).

WVP of the film was found to increase with increasing concentration of PDC developed at both pH conditions (Table 1). There are reports of increased WVP with increasing concentration of fish myofibrillar protein (14, 23, 24) similar to the present study. Increasing concentrations of protein leads to higher film thickness, which has positive correlation with WVP of films (31). Higher WVP was found in films developed under alkaline conditions as compared to films developed under acidic conditions. A previous report also showed similar results in case of films developed using tilapia fish myofibrillar protein isolate. The authors reported that films developed under acidic conditions have a stronger network with stronger interactions among protein molecules and high compactness, which might explain the lower WVP and higher TS of these films compared with films developed under alkaline conditions (32).

### 4.3 Protein solubility of films in different solvents

The films were dispersed in different solvents to reveal the interactive forces (ionic, hydrogen, hydrophobic, and disulfide bonds) involved in the formation of the three-dimensional network of protein molecules in film. Solvent b disrupts the electrostatic interactions, solvent c disrupts the hydrogen bonds and hydrophobic interactions, while solvent d can disrupt hydrogen bonds, hydrophobic interactions, and disulfide bonds (17). The protein solubility of films and PDC in the above-mentioned solvents is shown in Figure 1b, whereas Figure 1c shows the protein solubility of films in above-mentioned solvents with PDC concentration. Under acidic conditions, films prepared at all PDC concentrations showed no significant differences (*p* > 0.05) in protein solubility in solvent a and b. The same observation was found in case of PDC. This suggests electrostatic interaction has no significant role on PDC and films prepared under acidic conditions. The films prepared under alkaline conditions showed comparatively higher (p < 0.05) solubility in solvent a as compared to PDC and films prepared under acidic conditions. Films prepared at alkaline conditions with 0.5 and 1.5% PDC showed higher solubility in solvent a than solvent b in comparison to film prepared with 1% PDC. Therefore, in 1% PDC (pH 11), 3.3% protein resulted in electrostatic interaction. Higher solubility in solvent a as compared to solvent b in 0.5 and 1.5% PDC (pH 11) may be due to the effect of salting out (17). In solvent c, where urea and SDS are present, all films as well as PDC showed higher solubility (p < 0.05) as compared to solvent a and solvent b. Film prepared with 0.5, 1, and 1.5% PDC under acidic conditions showed 29.1, 16.6, and 31.8% proteins while, under alkaline conditions, 7.2, 11.1, and 16.0% protein, respectively, involved in hydrophobic and hydrogen bond interactions. In the case of PDC, 17.9% of proteins were involved in these interactions. For solvent d, where 2-mercaptoethanol was present, significantly higher protein solubility was found for PDC and for films developed under acidic conditions compared with films developed under alkaline conditions. Films developed using 0.5, 1, and 1.5% PDC under acidic conditions showed 35.6, 34.4, and 37.4% proteins involved in disulphide interaction, respectively, and 76.4% of proteins in case of PDC. Hence, disulphide bonds play a major role in these samples. There was no significant (p > 0.05) difference in solubility between solvent c and solvent d in case of films prepared under alkaline conditions; hence, disulphide interactions play no role in these films. Besides these, an insoluble fraction was present in each sample which did not solubilize in solvents used in the present study. Films with 0.5, 1, and 1.5% PDC developed in acidic conditions showed 35.4, 48, and 30.1% insoluble fraction, respectively. Whereas films with 0.5, 1, and 1.5% PDC developed in alkaline conditions showed higher insoluble fraction with value of 87.4, 81.7, and 76.2%, respectively. PDC had only 3% insoluble fractions. This suggests the presence of insoluble macromolecules or protein aggregates during film formation. Another reason may be due to the formation of other covalent bonds beside disulphide bonds during film synthesis as mentioned by previous authors (17). Under acidic conditions, protonation of charged amino acids in myofibrillar protein causes severe unfolding leading exposure of interior amino acids (28). This leads to the development of a higher number of new interactions, mainly disulphide bonds during drying of film-forming solution developed under acidic conditions. Hence, film developed under acidic conditions had higher TS and lower WS as compared to films developed under alkaline conditions.

### 4.4 Color analyses of films

The effect of storage on the color properties of film was studied for 15 days, and results are shown in Table 2. There was no significant effect (p > 0.05) of pH and PDC concentration on lightness (L*) found on day 0 (Table 2). However, a storage decrease in L* was found in films developed using 0.5% PDC under acidic condition and all films developed under alkaline condition, but films developed with 1 and 1.5% PDC developed under acidic condition showed no effect on L* with storage similar to film developed using shrimp muscle protein (33). However, a previous study showed decrease in L* with increasing concentration of tilapia fish myofibrillar protein in contrast to present study (14). This difference might be due to the use of different fish in the present study. An increase in redness (a*) at acidic pH with a decrease in redness at alkaline pH was observed with increasing concentration of PDC on day 0 (Table 2). Higher a* was found at acidic pH as compared to alkaline pH at similar PDC concentrations (Table 2). An increase in redness was found with storage at both pH conditions. A previous report showed decrease in a* in films developed using bigeye snapper fish surimi in both alkaline and acidic condition (23). The difference may be due to differences in fish species and film preparation methods in the present study. An increase in yellowness (b*) was observed in both acidic and alkaline conditions with increasing concentrations of PDC (Table 2). There was no significant difference (*p* > 0.05) in b* between films developed using 0.5% PDC at both pH conditions. However, at 1 and 1.5% PDC, film developed under alkaline conditions had higher b* as compared to film developed under acidic conditions on day 0. A previous report also showed higher yellowness in films developed under alkaline conditions as compared to acidic conditions. This is because alkaline pH is more effective in enhancing the Maillard reaction and favors the reductone formation from the Amadori products, leading to higher color development (27).

The yellowness of films increased with storage at both pH conditions (Table 2). A previous report also showed an increase in yellowness with increasing protein concentration similar to the present study in both acidic and alkaline condition (23). The reason for an increase in b* with storage is due to lipid oxidation mediated by haemoglobin which causes the formation of carbonyl compound followed by Maillard reaction with amino group of free amino acid, peptide and protein (27).

### 4.5 Surface micrograph of film

Film with 1% PDC developed under acidic conditions showed higher TS as compared to other films. Therefore, this film was subjected to scanning electron microscopy to observe the surface micrograph. For comparison, film with 1% PDC developed under alkaline conditions was also used. The SEM image of films developed under acidic and alkaline pH are shown in Figures 2a,b, respectively. It was found that film developed in acidic conditions had a smooth and homogenous structure as compared to film developed under alkaline conditions. As mentioned in a previous report, acidic pH might favor the solubilization and subsequent alignment of protein molecules which lead to stronger interaction of protein molecules with high compactness and homogenous structure. Due to this, films developed using red tilapia myofibrillar protein isolate under acidic conditions had higher TS and lower WVP and SEM micrograph showed smoother structure as compared to films developed under alkaline conditions (32), similar to the present study.

### 4.6 Effect of temperature on WS and WVP of film

Film developed using 1% PDC under acidic and alkaline conditions were also analyzed to check the effect of temperature on WS and WVP; results are shown in Figures 2c,d respectively. It was found that there was an increase in WS and WVP as the incubation temperature increased. It was also observed that, at each temperature, films developed under acidic conditions had lower WS and WVP as compared to films developed under alkaline conditions. A previous report also showed higher WS and WVP of films developed under alkaline conditions as compared to film developed under acidic conditions (34). A higher WVP of films with increasing temperature was also reported earlier (35).

A deviation in pH toward extreme acidic or alkaline conditions leads to protonation and deprotonation, respectively, of charged amino acids, which causes repulsion and, hence, unfolding of proteins, which disturbs their secondary and tertiary structure. In myofibrillar proteins, the *α*-helical structure is present in its native form, however, this structure reduced under both acidic and alkaline conditions, although more so under acidic conditions, hence the high exposure of different interior amino acids under acidic conditions (28, 36). This will help in the formation of high interactions such as hydrogen, hydrophobic, and disulphide bonds during drying process of film-forming solution under acidic conditions which lead to formation of films with higher tensile strength, and lower WS, and WVP in present study.

The EB, TS, and WS of the developed film were 146.75%, 6.86 MPa, and 28.1%, respectively. Previously, it was mentioned that if EB < 20%, then the film is brittle (37). As the developed film has very high EB, it is not brittle. The film also has better TS as compared to previously developed films. For instance, film developed using poly (butylene adipate-co-terephthalate) with polylactide had TS and EB of 1.35 MPa and 5.63%, respectively (38). The TS of film developed using unwashed basa fish waste was 0.8 MPa (9). Film developed using brea gum had a comparable TS of 7.5 MPa (39). The developed film had lower WS as compared to previously developed biodegradable films. For instance, film developed using unwashed base fish myofibrillar protein was 65% (9) and 100% WS was found in film developed using brea gum (39). Hence, developed films have higher TS and EB and lower WS as compared to previous developed films and has wider applications in food industry. These properties make it suitable for flexible food packaging applications such as pouches and wraps, as well as coating layers for dry or low-moisture food products including snacks, bakery items, and confectionery. The film’s partial water solubility allows for potential use in edible or compostable packaging, while its low water vapor permeability helps extend the shelf life of moisture-sensitive foods.

## 5 Conclusion

Films prepared under acidic conditions exhibited higher tensile strength, lower water solubility, lower water vapor permeability, and less yellowness compared to those developed under alkaline conditions, with the film containing 1% protein concentrate at pH 2.8 demonstrating the most favorable properties among all formulations. Therefore, this approach offers a practical way to develop biodegradable films from Basa fish waste, reducing environmental pollution while promoting the sustainable utilization of fishery by-products. These findings also provide a foundation for scaling up the production of fish waste-derived biodegradable films and evaluating their performance in real food packaging systems, paving the way for sustainable alternatives to conventional plastics in the food industry.

## Data Availability

The original contributions presented in the study are included in the article/supplementary material, further inquiries can be directed to the corresponding authors.
